# The Evolution of Ischemia‐Reperfusion Injury Research in Ischemic Stroke: Insights From a Two‐Decade Bibliometric Analysis

**DOI:** 10.1002/brb3.70445

**Published:** 2025-04-01

**Authors:** Hongyu Xu, Xinglin Lu, Rongxing Qin, Lingduo Shao, Li Chen

**Affiliations:** ^1^ Department of Neurology the First Affiliated Hospital of Guangxi Medical University Nanning China; ^2^ Department of Critical Care Medicine Affiliated Minzu Hospital of Guangxi Medical University Nanning China

**Keywords:** bibliometric analysis, hot spots, ischemia‐reperfusion injury, ischemic stroke, trends

## Abstract

**Background:**

Ischemic stroke is a complex disease with high mortality and disability rates. Ischemia‐reperfusion injury is a common aftermath. There have been significant advancements in understanding ischemia‐reperfusion injury in ischemic stroke over the past two decades. This study aims to evaluate the current state of ischemia‐reperfusion injury in ischemic stroke through bibliometric analysis, identifying key research areas and emerging trends.

**Methods:**

Relevant documents in the Web of Science Core Collection, SCI‐Expanded from January 1, 2003, to December 31, 2023, were downloaded on July 10, 2024. Bibliometric analysis was performed using HistCite, VOSviewer, CiteSpace, and Bibliometrics online analysis platform.

**Results:**

A total of 2179 research papers from 611 journals in 66 countries were included in this study. Among these papers, China emerged as the leading contributor of ischemia‐reperfusion injury in ischemic stroke publications, with Capital Medical University standing out as the institution with the highest number of publications in this area. Y. Zhang was identified as the author with the most publications during the study period. *Brain Research* was found to be the most prolific journal for this research. The keywords “ferroptosis”, “circular RNA”, “polarization”, and “fatty acid binding protein” represent the current hot spots of ischemia‐reperfusion injury in ischemic stroke research.

**Conclusion:**

This bibliometric analysis offers the first thorough overview of hot spots and research trends in ischemia‐reperfusion injury in ischemic stroke over the previous 21 years, providing researchers with new ideas in the field. “ferroptosis”, “circular RNA”, “polarization”, and “fatty acid binding protein” may be the focus of future studies.

## Graphical abstract

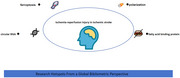

## 1 Introduction

Thrombosis or embolism‐induced disruption of cerebral blood flow is the etiology of ischemic stroke (Qin et al. 2022). An estimated 13.7 million ischemic stroke cases occur worldwide annually, with 5.5 million of those instances ending in death (GBD 2016 Stroke Collaborators 2019). Stroke was also the second most common cause of disability (GBD 2019 Diseases and Injuries Collaborators 2020). Taking this into account, the cornerstone of all present‐day therapeutic methods for ischemic stroke is the restoration of blood flow and revascularization as soon as possible (Kalogeris et al. 2012). For acute ischemic stroke, intravenous thrombolysis is the recommended course of treatment (Khandelwal et al. 2016), and mechanical thrombectomy has demonstrated encouraging outcomes in vascular recanalization, especially in cases of major artery blockage (Powers et al. 2019). However, only 37% of patients who got this intervention were able to become independent in their daily lives (Wollenweber et al. 2019).

Ischemia‐reperfusion injury occurred in up to 54% of ischemic stroke patients with reperfusion (Zhou et al. 2023). Hence, not all stroke patients will benefit from restored blood flow. Furthermore, it appears evident that reperfusion can also set off pathogenic processes that worsen damage from ischemia itself and may cause tissue damage in distal organs due to the release of mediators into the bloodstream and subsequent transport to distal organs, even though it is necessary to restore the delivery of oxygen and nutrients to support cellular metabolism and remove potentially harmful byproducts of cellular metabolism (Kalogeris et al. 2012). Researchers from all around the world have therefore focused a lot of emphasis on ways to lessen ischemia‐reperfusion harm in ischemic stroke. Conducting a thorough analysis of the advancements in research and development trends related to ischemia‐reperfusion injury in ischemic stroke is therefore imperative.

Through the evaluation of publication quantity and quality, bibliometric analysis makes it easier to examine scholarly literature within a particular academic discipline (Ninkov et al. 2021; Arruda et al. 2022). Researchers can use this method to identiy the countries/regions of production, the institutions and authors engaged, as well as research priorities and current concerns in the field (Glanzel et al. 2019; Thompson and Walker 2015). As of right now, there is a lack of documented bibliometric analysis on ischemia‐reperfusion injury in ischemic stroke.

Therefore, the purpose of this paper is to sort out the research status of ischemia‐reperfusion injury of ischemic stroke through multiple combination software, and visually and comprehensively analyze the new trends and frontiers in this field.

## 2 Methods

### 2.1 Data Source and Search Strategy

**FIGURE 1:**
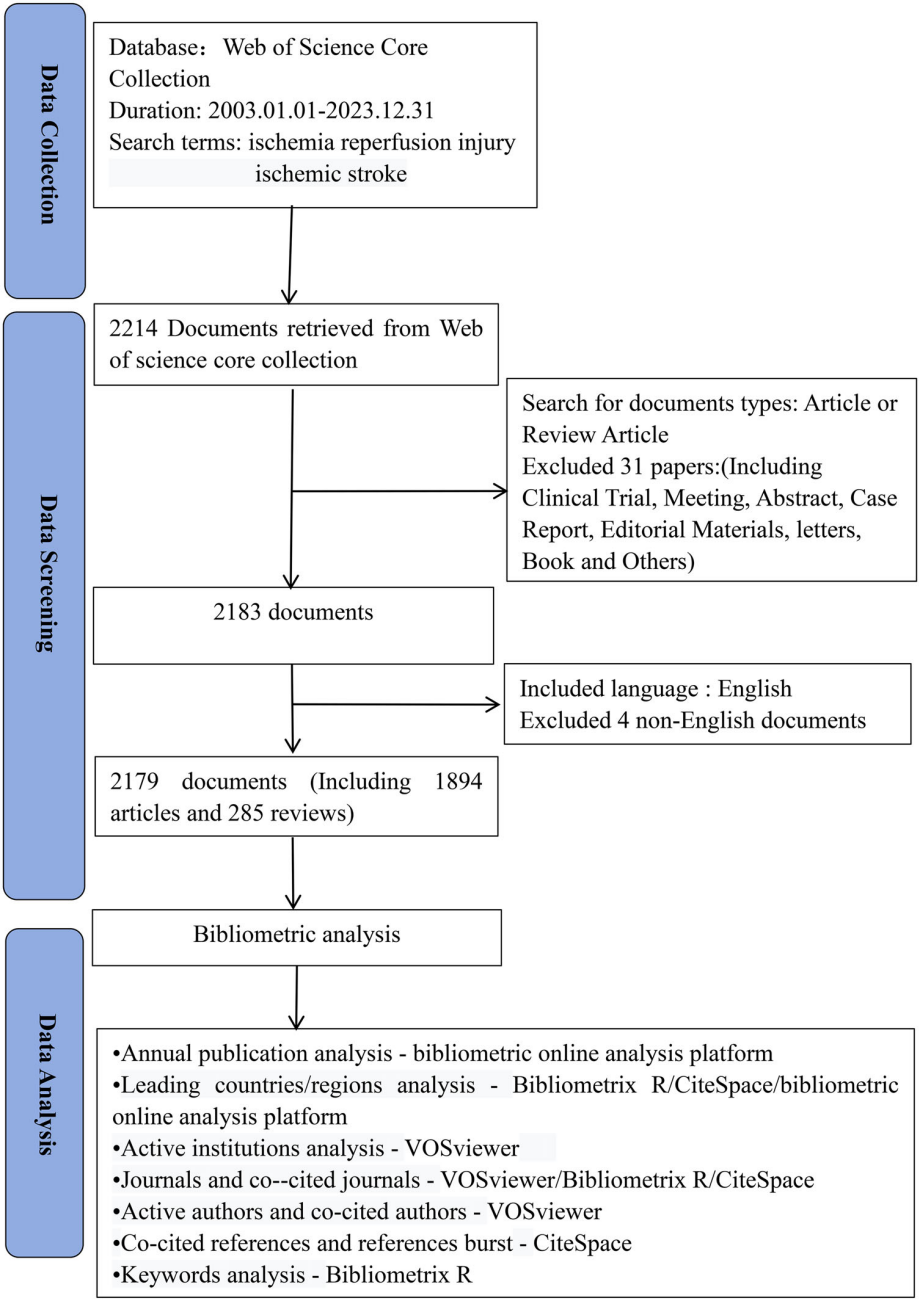
The detailed process of screening and enrollment is displayed.

The Science Citation Index Expanded Web of Science Core Collection (WOSCC) is recognized globally as the most authoritative database of scientific and technological literature, making it the database of choice for bibliometric analysis due to its high credibility and careful review standards. The WoSCC was utilized to retrieve pertinent studies on ischemia‐reperfusion injury in ischemic stroke from January 1, 2003, to December 31, 2023. All data collection was carried out on July 10, 2024, to avoid omissions due to rapid database updates and thus maintain data consistency. The search strategy is detailed in Table S1. Included studies were mainly in English with a focus on selected articles and reviews. Fu et al. (2012) have suggested that more accurate and comprehensive bibliometric results can be obtained by the “front page” including paper titles, abstracts, and author keywords. The search keywords “ischemia‐reperfusion injury” and “ischemic stroke” were searched with the title (TI), abstract (AB), and author keyword (AK) fields in advanced search on WoSCC. A total of 2179 documents were ultimately incorporated into the study, with all the eligible data exported in the formats of Plain text file, Tab delimited file, and BibTex, containing complete records and references. Figure 1 provides the detailed process of screening and inclusion.

### 2.2 Data Visualization and Analysis

Because of the characteristics of various bibliometrics software, we used multi‐software to conduct a comprehensive analysis of the selected documents, which can play a complementary role.

Using the traceability of citations, HistCite is a tool that can discover important literature in particular domains, analyze literature citations, and create an overall picture of citations across literatures in addition to an index of citations of a single document. The literature citation analysis in our study was conducted using HistCite Pro 2.1 (Garfield et al. 2006), which was utilized to calculate the total local citation score (TLCS), total global citation score (TGCS), and citation records for publications, journals, countries/regions, institutions, and authors.

Researchers can extract crucial information from a variety of publications by using VOSviewer, which uses a probability‐theory‐based method for data standardization (van Eck and Waltman 2009; Yeung and Mozos 2020). Building cooperative, co‐citation, and co‐occurrence networks is a typical use for this software in a variety of scientific fields (Jia and Mustafa 2022). It is especially noticeable in the visual analysis of keywords and author collaboration. In our study, VOSviewer (1.6.20) was used to identify high‐productivity journals, institutions, and authors, in addition to major co‐cited journals, and authors, while also constructing visual networks related to the data. The size of the nodes indicated the frequency of citations, and the co‐occurrence link was indicated by the connections between the nodes.

Professor Chen created CiteSpace, a software tool for bibliometric analysis and visualization intended for research data exploration (Glanzel et al. 2019; Chen 2020). In our work, CiteSpace (version 6.3.R1) produced a dual map overlay that made it easier to see how different journals within our research subject are connected to one another. In addition, visualizations and analyses were performed on networks of countries/regions and co‐cited references. Research themes related to ischemia‐reperfusion injury were also identified by cluster analysis of co‐cited references. Finally, CiteSpace was utilized to detect bursts in the strength of references, aiding in the identification of significant hot spots and frontiers within ischemia‐reperfusion injury in ischemic stroke.

Bibliometrix is a comprehensive R language package for bibliometric analysis and scientific visualization of literature, developed by Massimo Aria and Corrado Cuccurullo (Aria and Cuccurullo 2017). Bibliometrix R packages (4.4.0) were employed for the analysis of the country collaboration map, corresponding author's countries, word cloud of keywords, keywords co‐occurrence network, trend topics, and core journals. Notably, the core journal analysis was based on Bradford's law, which utilizes the Bradford law to describe the distribution of citations in a subject or field (Venable et al. 2016).

An additional tool for bibliometric analysis of citation data is the bibliometric online analysis platform (http://bibliometric.com). It is excellent at analyzing the cooperative relationship between countries and the number of articles in each country over the years.

## 3 Results

### 3.1 Annual Publication Analysis

**FIGURE 2:**
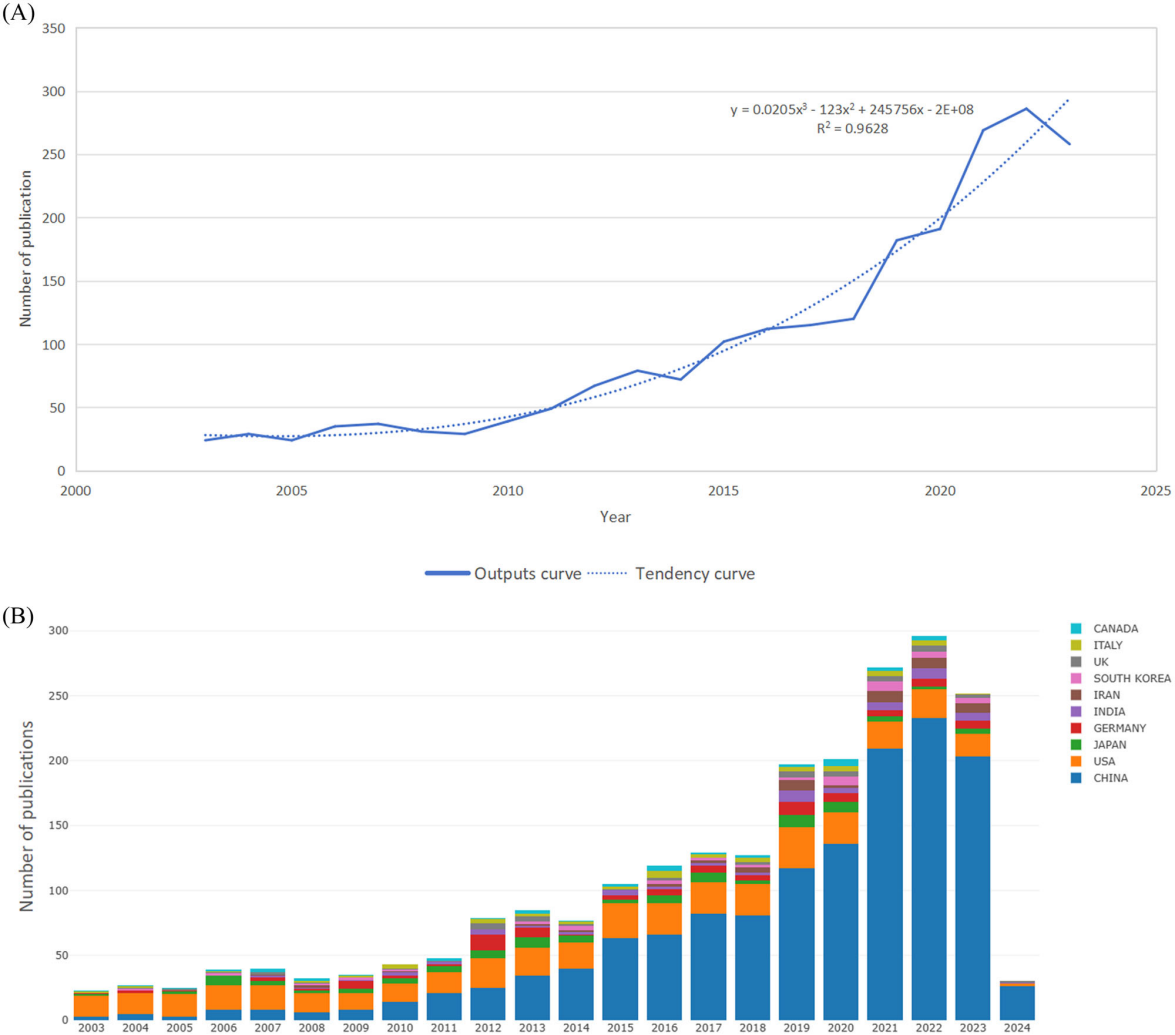
Annual and number of publications from 2003 to 2023 (A); Number of publications per year by country (B).

Based on our search approach, a total of 2179 papers linked to ischemia‐reperfusion injury in ischemic stroke, comprising 1894 “articles” and 285 “reviews”, were identified in the WOSCC database from 2003 to 2023. The yearly publication counts related to ischemia‐reperfusion injury are shown in Figure 2A, which shows a generally increasing trend despite fluctuations in yearly publication numbers. From 2003 to 2011, the field of ischemia‐reperfusion injury was characterized by the early embryonic stage, with an average of about 30 publications per year and no more than 50 publications annually. From 2012 to 2023, there was a significant increase in the number of publications within the field of ischemia‐reperfusion injury, growing from 67 to 258 (Table S2). This indicates that researchers are placing an increasing emphasis on studying ischemia‐reperfusion injury. The multiple regression curve of the annual number of documents showed that *R* ^2^ = 0.9628, indicating a strong correlation with the trend, and further indicating that ischemia‐reperfusion injury has experienced substantial growth and progress. As shown in Figure 2B, the annual number of published papers by each country shows that China has caught up to the United States in this field of study, which was initially conducted by the former. China is the country that publishes the most papers annually, by a wide margin.

### 3.2 Leading Countries/Regions Analysis

Between 2003 and 2023, 66 countries/regions have made contributions to the scholarly discussion on ischemia‐reperfusion injury. Table 1 displays the top 10 countries/regions ranked based on the number of published papers in this domain. China led with 1346 papers, followed by the USA with 427 papers and Japan with 92 papers. China received the highest number of citations (TGCS = 41,473), followed by USA (TGCS = 34,174), and Germany (TGCS = 9292). Figure 3A depicts a map showing countries/regions’ collaboration on ischemia reperfusion injury in the field of ischemic stroke. The red line showed that there was some type of cooperation between two countries/regions, with the thickness of the lines reflecting the amount of collaboration. Figure 3B shows that China's Single Country Publication (SCP) and Multiple Country Publication (MCP) are the largest, followed by the USA. The proportion of MCP in other countries is quite low, and MCP reflects a country's international exchange and collaboration. As the graphic shows, international cooperation in this area still has to be strengthened. The frequency of exchanges and cooperation between countries is shown in Table S3. Following that, we used CiteSpace and Bibliometrics online analysis platform to display international cooperation and create the cooperation network illustrated in Figure 3C,D. The top three countries in terms of cooperation intensity were China, USA, and Germany.

**TABLE 1:** Top 10 productive countries and most cited countries.

	Most productive countries					Most cited countries				
Rank	Country	Records	TLCS	TGCS	H‐Index	Country	Records	TLCS	TGCS	H‐Index
1	Peoples Republic of China	1346	1633	41473	83	Peoples Republic of China	1346	1633	41473	83
2	USA	427	927	34174	42	USA	427	927	34174	42
3	Japan	92	213	9097	39	Germany	87	201	9292	34
4	Germany	87	201	9292	34	Japan	92	213	9097	39
5	India	60	46	1270	23	Australia	28	127	6404	20
6	UK	50	50	3268	28	Argentina	2	30	4105	2
7	Iran	48	33	953	17	UK	50	50	3268	28
8	Taiwan	47	71	2275	23	Italy	44	38	2367	24
9	South Korea	45	41	1486	19	Taiwan	47	71	2275	23
10	Italy	44	38	2367	24	Canada	39	46	1520	23

Abbreviations: TGCS: total global citation score; TLCS: total local citation score.

FIGURE 3Country/region collaboration world map of ischemia reperfusion injury research in ischemic stroke (A). Corresponding Author's Countries of ischemia reperfusion injury research in ischemic stroke (B). The network map of countries/regions for ischemia reperfusion injury research in ischemic stroke (C, D).

**Figure:**
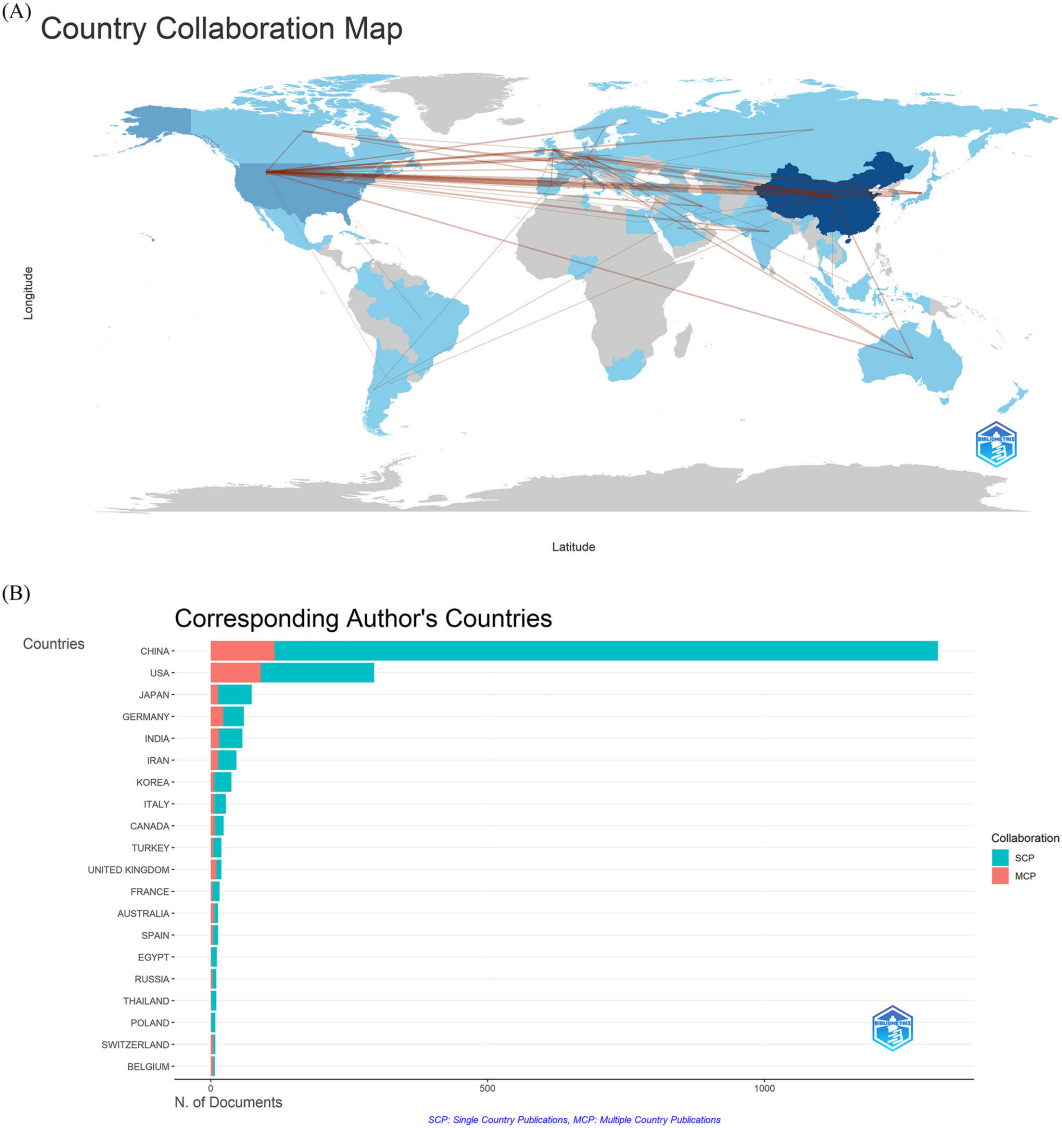

**Figure:**
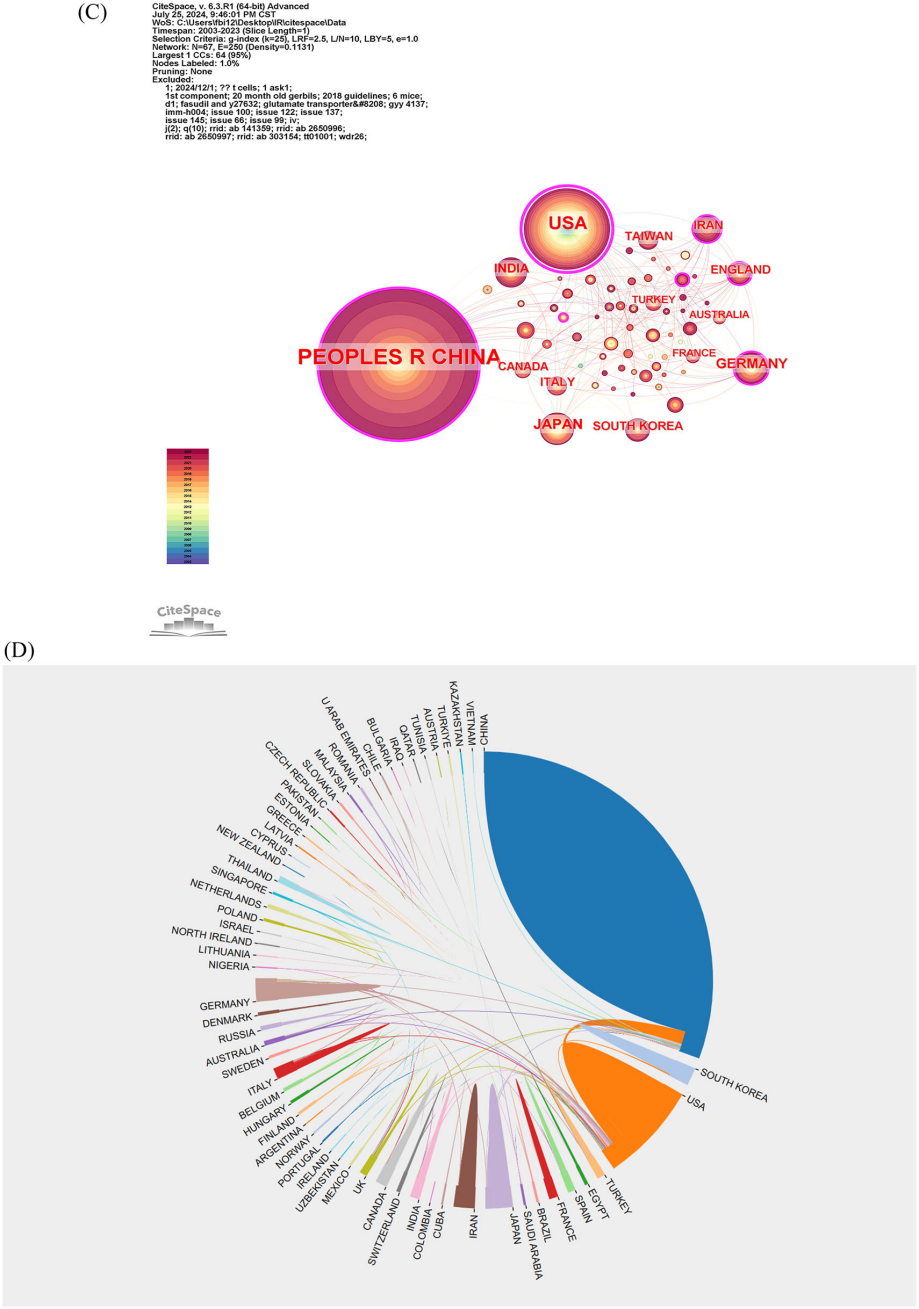

### 3.3 Active Institutions Analysis

**FIGURE 4:**
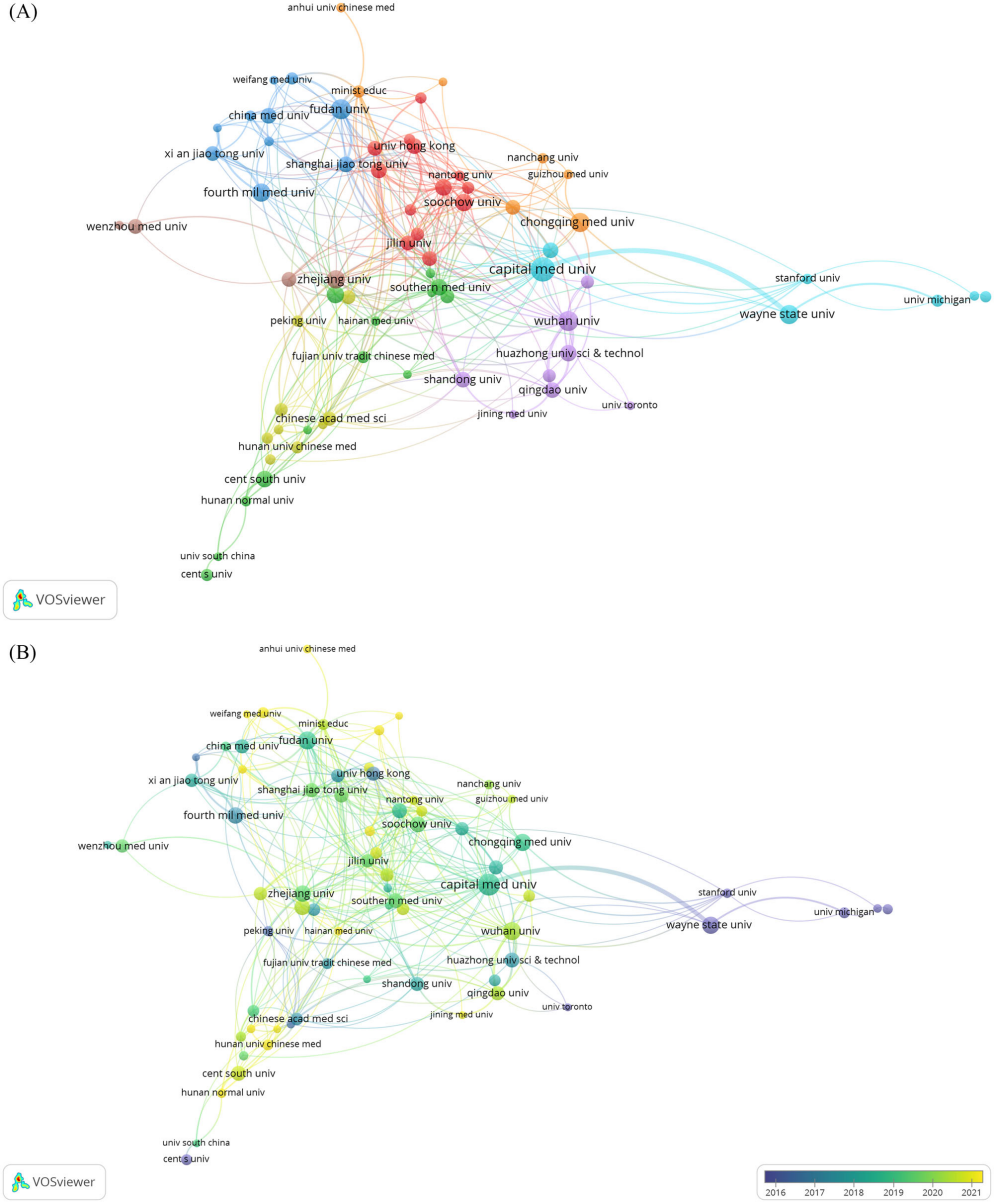
Graphical representation of collaboration among institutions. Network clustering (A); Time‐overlapping network (B).

A total of 2105 institutions were involved in the ischemia‐reperfusion injury in ischemic stroke study, Table 2 highlights the top 10 institutions based on the number of published papers, indicating their prominence in the field. It is noteworthy that nine out of the ten most productive organizations are located in China. Leading the pack was Capital Medical University, with 69 papers. Following closely behind was Wuhan University, tied for second place with 47 papers. Fudan University secured third place by publishing 46 papers. Capital Medical University exhibits the highest TGCS and H‐index. While most of the top 10 most prolific institutions are from China, only one of the top 10 most‐cited institutions is from China. The top three most cited institutions are Stanford University, the University of Melbourne, and the University of Pittsburgh. Collaborative networks were built using VOSviewer in order to learn more about inter‐institutional collaboration. Figure 4A illustrates institutional collaboration; the thickness of the lines indicates the degree of intensity of collaboration between two universities. Capital Medical University, Wayne State University, Stanford University, Zhejiang University, Shangdong University, and Fudan University also collaborate to a considerable extent. A thorough examination of institutional relationships over time is presented in Figure 4B, which shows that in 2016, Wayne State University, Stanford University, and the University of Michigan were the main centers of research for ischemia‐reperfusion injury in ischemic stroke. On the other hand, following 2019, Wuhan University, Hunan University Chinese Medicine, Capital Medical University, and Chongqing Medical University all demonstrated an increased involvement in this area.

**TABLE 2:** Top 10 most productive and cited institutions.

	Most productive institutions					Most cited institutions						
Rank	Institutions	Country	Records	TLCS	TGCS	H‐Index	Institutions	Country	Records	TLCS	TGCS	H‐Index
1	Capital Medical University	China	69	105	1744	26	Stanford University	USA	15	77	5448	15
2	Wuhan University	China	47	49	1615	23	University of Melbourne	Australia	8	92	5062	8
3	Fudan University	China	46	35	978	22	University of Pittsburgh	USA	8	37	4801	8
4	Chongqing Med University	China	44	68	1461	19	Columbia University	USA	12	63	4614	10
5	Wayne State University	USA	42	148	2146	23	University of Wurzburg	Germany	9	50	4595	12
6	Fourth Military Medical University	China	40	76	1449	19	University of Texas Health Science Center San Antonio	USA	6	44	4396	7
7	Zhejiang University	China	39	35	1105	20	Technische Universität Dresden	Germany	3	42	4203	6
8	Soochow University	China	37	35	962	16	Memorial Sloan Kettering Cancer Center	USA	2	30	4191	6
9	Zhengzhou University	China	37	24	1094	16	Guangzhou Medical University	China	7	41	4185	6
10	Huazhong University Science & Technology	China	34	85	1396	18	University of Arizona	USA	6	33	4176	5

### 3.4 Journals and Co‐Cited Journals

**FIGURE 5:**
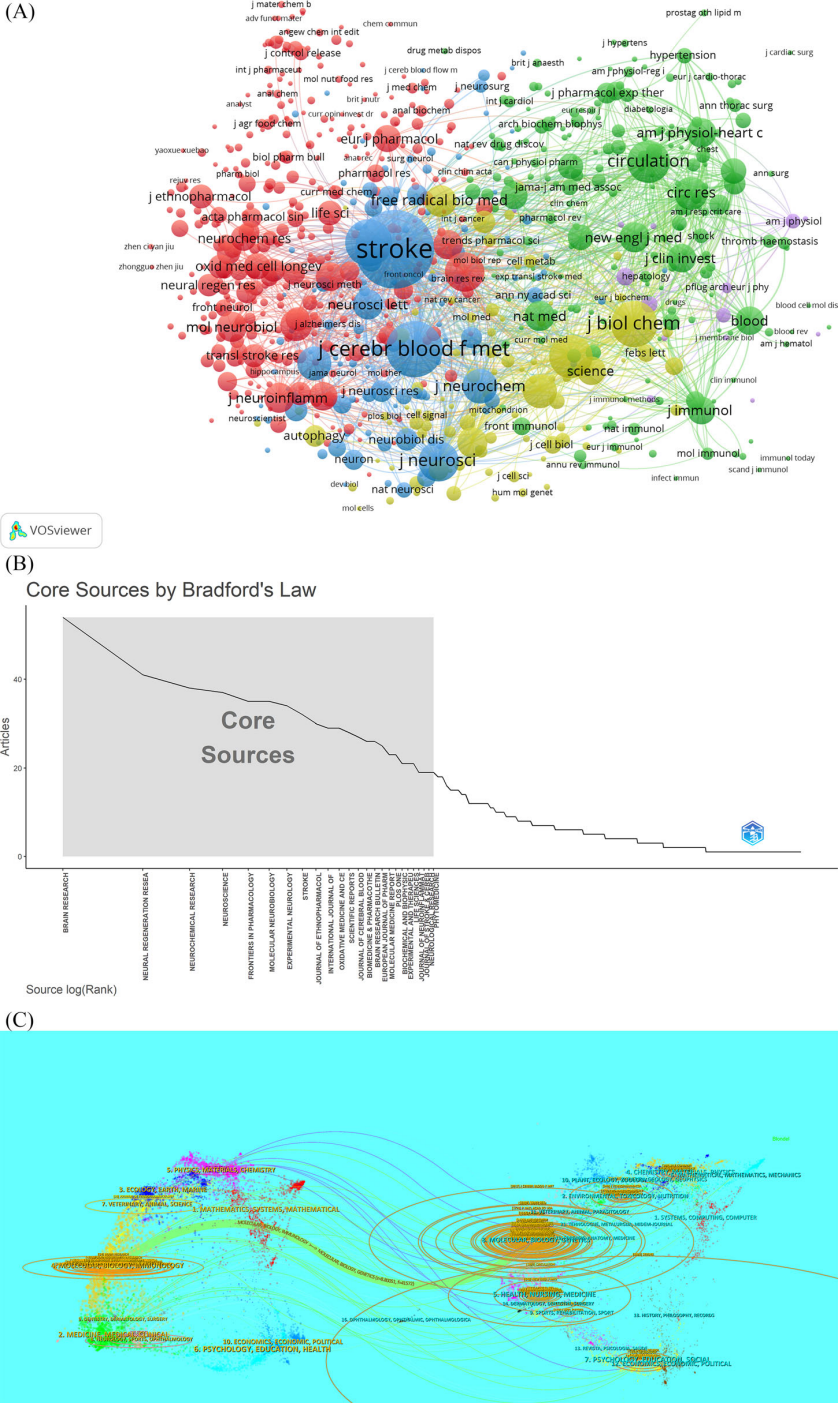
Analysis of journals involved in ischemia reperfusion injury research in ischemic stroke. The visualization of journal co‐citation analysis (A); According to the Bradford's law, the top 25 core journal (B); The dual‐map overlay of journals on ischemia reperfusion injury research (C).

Research on ischemia‐reperfusion injury has been published in 611 scientific journals. The top 10 journals and co‐cited journals in ischemic stroke research on ischemia‐reperfusion injury are shown in Table 3. Among these, *Brain Research* has the highest number of publications related to ischemia‐reperfusion injury in ischemic stroke (*n* = 54), followed by *Neural Regeneration Research* (*n* = 41) and *Neurochemical Research* (*n* = 39). Even though these journals publish a substantial amount of papers, there are surprisingly few journals with an impact factor (IF) higher than 10. Among the top 3 most cited journals were *Cell* (TGCS = 4661), *Nature Medicine* (TGCS = 3229), and *Stroke* (TGCS = 3165). Then, journals with a co‐citation frequency of at least 20 were selected for network mapping (Figure 5A), with the size of the node indicating the co‐citation frequency. Notably, the journals *Stroke* and *Journal of Cerebral Blood Flow and Metabolism* exhibited the largest nodes, signifying an enormous amount of co‐citations. Based on the number of publications, journals in a given subject can be divided into core, related, and non‐related journals according to Bradford's law; each category usually accounts for one‐third of the total number of publications. Based on Bradford's law, Figure 5B shows the top 25 core journals in this discipline. The top three core journals are *Brain Research*, *Neural Regeneration Research*, and *Neurochemical Research*.

**TABLE 3:** Top 10 productive journals and top 10 most cited journals.

Rank	Most productive journals	Records	TGCS	IF (2023)	Publishers	Most cited journals	Records	TGCS	IF (2023)	Publishers
1	*Brain Research*	54	2164	2.7	Elsevier	*Cell*	2	4661	45.5	Cell Press
2	*Neural Regeneration Research*	41	763	5.9	Wolters Kluwer Medknow Publications	*Nature Medicine*	3	3229	58.7	Springer Nature
3	*Neurochemical Research*	39	801	3.7	Springer US	*Stroke*	32	3165	7.8	Lippincott Williams and Wilkins Ltd.
4	*Molecuar Neurobiology*	38	1795	4.6	Springer US	*Journal of Cerebral Blood Flow and Metabolism*	27	2409	4.9	SAGE Publications Inc.
5	*Neuroscience*	38	1271	2.9	Elsevier Ltd	*Brain Research*	54	2164	2.7	Elsevier
6	*Journal of Ethnopharmacoloy*	36	852	4.8	Elsevier Ireland Ltd	*Molecular Neurobiology*	38	1795	4.6	Springer US
7	*Frontiers in Pharmacology*	35	693	4.4	Frontiers Media S.A.	*Proceedings of the National Academy of Sciences of the United States of America*	8	1474	9.4	National Academy of Sciences
8	*Experimental Neurology*	34	1191	4.6	Academic Press Inc.	*International Review of Cell and Molecular Biology*	1	1474	9.4	Elsevier Inc.
9	*Stroke*	32	3165	7.8	Lippincott Williams and Wilkins Ltd.	*Neuroscience*	38	1271	2.9	Elsevier Ltd.
10	*International Journal of Molecular Science*	29	581	4.9	MDPI (Basel, Switzerland)	*Experimental Neurology*	34	1191	4.6	Academic Press Inc.

A dual‐map overlay, as shown in Figure 5C, shows many inter‐domain connections between journals: the left side of the map shows citing journals, and the right side shows cited journals. Labels on both sides indicate the different disciplines that each journal covers. The colored lines connecting the two sides show citation paths, which reveal two different reference paths. It is worth noting that the majority of published articles were published in journals that fall under the categories of molecules, biology, and immunology, while the majority of cited publications fell under the categories of molecules, biology, and genetics.

### 3.5 Active Authors and Co‐Cited Authors

The top 10 authors each have more than 30 publications published, out of the 9467 authors who have published on ischemia‐reperfusion injury studies in ischemic stroke. As shown in Table 4, all of these authors are affiliated with institutions in China. Among these authors, Y. Zhang (*n* = 59, TGCS = 1628) stands out as the most prolific, his current research focuses on the anti‐mitochondrial apoptosis mechanism, regulating microglia polarization, ROS/inflammatory Microenvironment in cerebral ischemia‐reperfusion injury (Dong et al. 2023; X. Zhao et al. 2024; Zhang et al. 2024). Y. Wang (*n* = 55, TGCS = 1633), Li Y. (*n* = 42, TGCS = 883), L. Wang (*n* = 38, TGCS = 707), and Q. Wang (*n* = 37, TGCS = 1489) came after Y. Zhang. Table 4 also presents the top 10 cited authors, with A. I. Bush (*n* = 3, TGCS = 4581), A. Linkermann (*n* = 2, TGCS = 4223), D. D. Zhang (*n* = 2, TGCS = 4106), ranking among the top three.

**TABLE 4:** Top 10 productive authors and most cited authors on ischemia reperfusion injury in ischemic stroke.

	Most productive authors					Most cited authors				
Rank	Author	Records	TLCS	TGCS	Country	Co‐cited author	Records	TLCS	TGCS	Country
1	Zhang Y	59	67	1628	China	AI Bush	3	68	4581	Australia
2	Wang Y	55	63	1633	China	A Linkermann	2	30	4223	Australia
3	Li Y	42	31	883	China	D. D. Zhang	2	35	4106	China
4	Wang L	38	18	707	China	M. Conrad	2	30	4092	USA
5	Wang Q	37	85	1489	China	X. J. Jiang	2	30	4085	China
6	Chen J	36	40	1068	China	J. P. F. Angeli	1	30	4072	Germany
7	Liu Y	35	44	771	China	H. Bayir	1	30	4072	USA
8	Yang Y	32	23	1112	China	S. J. Dixon	1	30	4072	Germany
9	Zhang L	32	59	942	China	S. Fulda	1	30	4072	Germany
10	Li X	30	27	765	China	S. Gascón	1	30	4072	USA

**FIGURE 6:**
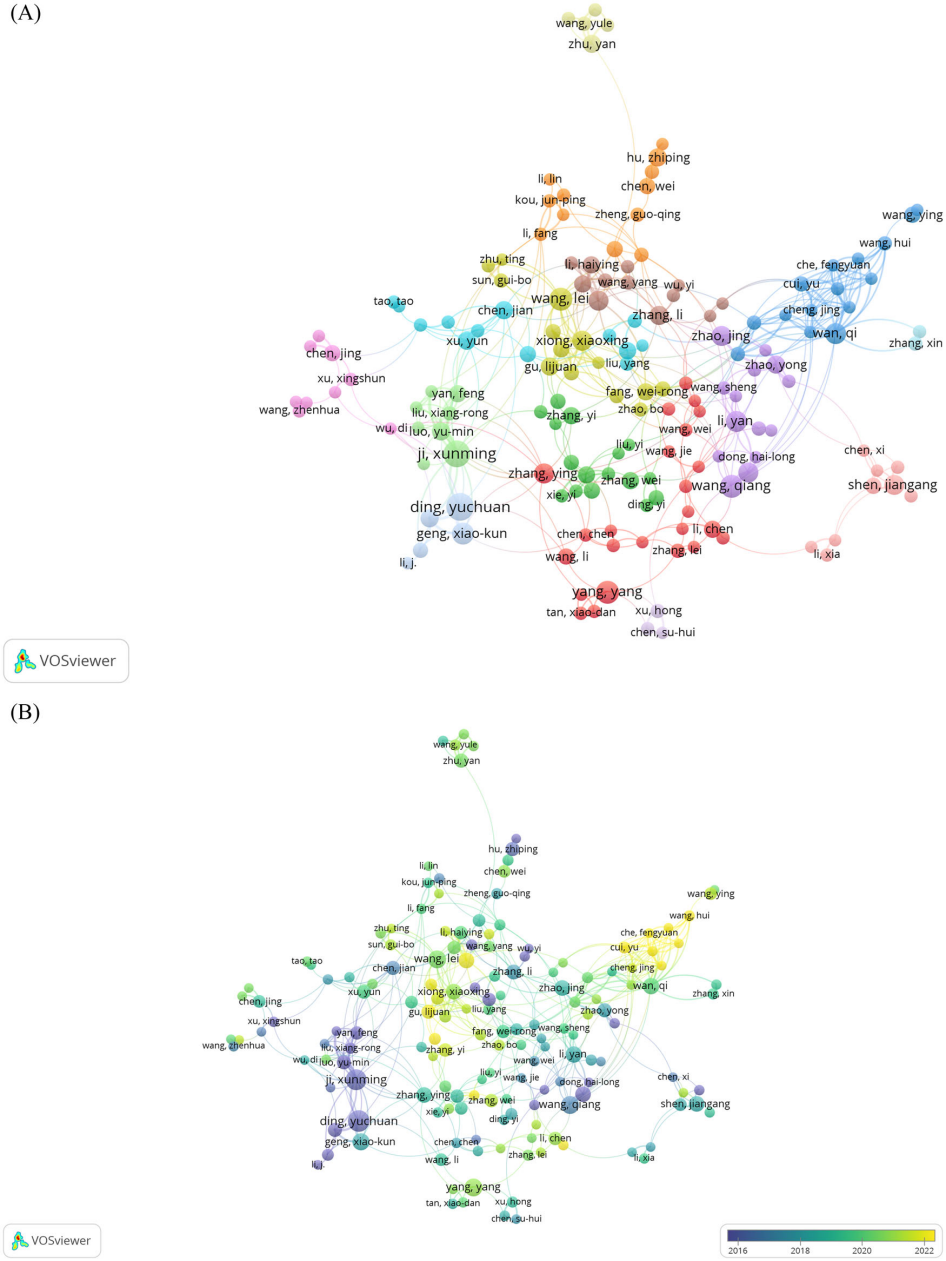
Graphical representation of collaboration among authors. Network clustering (A); Time‐overlapping network (B).

For an in‐depth study of author collaboration, we used VOSviewer to create an author network, focusing on authors with five or more articles (Figure 6). The color designates the authors' cluster, and the size of the circles denotes the total number of articles. An illustration of a collaboration network between multiple authors is provided in Figure 6A. Notable examples of this collaboration include the tight cooperation between Q. Wang, C. Ti, and Q. Wan, as well as the similarities between Y. Yang, X. D. Tan, Wang Li, and L. Zhang. Furthermore, X. M. Ji, Y. C. Ding, X. R. U, F. Yan, etc. were some of the earliest authors who investigated ischemia‐reperfusion injury studies in ischemic stroke, whereas F. Y. Che, Y. Cui, etc. are emerging authors from more recent years, as shown by the plot overlay with time shown in Figure 6B.

### 3.6 Co‐Cited References and References Burst

Table 5 lists the top 10 highly cited literature in the field (Kalogeris et al. 2012; Stockwell et al. 2017; Eltzschig and Eckle 2011; Gelderblom et al. 2009; Tang et al. 2007; Vaseva et al. 2012; Peng et al. 2019; Cadenas 2018; Łowicka and Bełtowski 2007; Chouchani et al. 2013), the majority of which are reviews. The most cited document, authored by Stockwell, BR (Stockwell et al. 2017) and published in *Cell*, titled “Ferroptosis: A Regulated Cell Death Nexus Linking Metabolism, Redox Biology, and Disease” (TGCS = 4072), reported the mechanics of ferroptosis, links to other fields of biology and medicine were highlighted, and resources and guidelines for researching this newly‐emerging type of controlled cell death were suggested in this primer. The second most cited document was authored by Eltzschig and Eckle (2011) (TGCS = 2399). Ranked third was written by Kalogeris et al. (2012) (TGCS = 1474).

**TABLE 5:** Top 10 most cited references.

Rank	Title	First author	Year	Journal	TGCS	Brief description	Document type /References
1	Ferroptosis: A Regulated Cell Death Nexus Linking Metabolism, Redox Biology, and Disease	B. R. Stockwell	2017	*Cell*	4072	The mechanics of ferroptosis were reviewed, links to other fields of biology and medicine were highlighted, and resources and guidelines for researching this newly‐emerging type of controlled cell death were suggested in this primer.	Review/24
2	Ischemia and reperfusion‐from mechanism to translation	H. K. Eltzschig	2011	*Nature Medicine*	2399	Deep tissue hypoxia and microvascular dysfunction were caused by an imbalance in the ischemic organ's metabolic supply and demand. The activation of innate and adaptive immune responses as well as cell death programs was further enhanced by subsequent reperfusion. The article discussed tissue inflammation and organ failure brought on by ischemia and reperfusion.	Review/25
3	Cell Biology of Ischemia/reperfusion Injury	T. Kalogeris	2012	*International of Cell and Molecular Biology*	1474	This treatise aims to give a thorough overview of the mechanisms that lead to I/R injury. From this, it should be clear that, in order to limit the extent of I/R injury, a combination of molecular and cellular approaches targeting multiple pathologic processes must be used to increase regenerative capacity and enhance resistance to cell death in order to effect long‐lasting repair of ischemic tissues.	Review/4
4	Temporal and Spatial Dynamics of Cerebral Immune Cell Accumulation in Stroke	M. Gelderblom	2009	*Stroke*	785	This work used a mouse cerebral ischemia‐reperfusion damage model to identify the temporal and geographical infiltration of immune cell types and their activation patterns.	Article/26
5	Pivotal role for neuronal Toll‐like receptors in ischemic brain injury and functional deficits	S. C. Tang	2007	*Proceedings of the National Academy of Sciences of the United States of America*	637	The article revealed that neurons express a variety of TLRs and that IFN‐gamma activation and energy deprivation cause an increase in TLR2 and TLR4 levels in neurons. The results identify a signaling route for TLR2 and TLR4 in neurons that promotes apoptosis and may make neurons susceptible to ischemia death.	Article/27
6	p53 Opens the Mitochondrial Permeability Transition Pore to Trigger Necrosis	A. V. Vaseva	2012	*Cell*	589	The study implicated the mitochondrial p53‐CypD axis in stroke pathology and found that it plays a significant role in oxidative stress‐induced necrosis.	Article/28
7	Mitochondrial dysfunction in atherosclerosis	N. R. Madamanchi	2007	*Circulation Research*	572	They linked this extensive amount of research to the clinical symptoms that predispose people to atherosclerosis and its complications in this review.	Review/29
8	ROS and redox signaling in myocardial ischemia‐reperfusion injury and cardioprotection	S. Cadenas	2018	*Free Radical Biology and Medicine*	533	This review's first section covered current research and debates about the mechanisms underlying the mitochondrial electron transport chain's production of superoxide after IR injury, as well as the role played by the NOX isoforms NOX1, NOX2, and NOX4 that are expressed in cardiomyocytes. The redox mechanisms of cardiomyocyte mitochondrial protection were examined in the second section. Ultimately, the conversation revolved around the clinical and translational consequences of these cardioprotective pathways.	Review/30
9	Hydrogen sulfide (H_2_S) ‐: the third gas for interest for pharmacologists	E. Lowicka	2007	*Pharmacological Reports*	516	Vascular tone, myocardial contractility, neurotransmission, and insulin production are all regulated by H2S. However, overproduction of H'S may have a role in the development of inflammatory illnesses, septic shock, cerebral stroke, and mental retardation in Down syndrome patients. Therefore, it may be beneficial to reduce H'S production in these conditions.	Review/31
10	Cardioprotection by S‐nitrosation of a cysteine switch on mitochondrial complex I	E. T. Chouchani	2013	*Nature Medicine*	495	The study investigated the cardioprotective effects of mitochondrial S‐nitrosation during the reperfusion phase of myocardial infarction in vivo in mice using the mitochondria‐selective S‐nitrosating chemical MitoSNO. The findings shown that blocking complex I reactivation through alteration of a cysteine switch is a strong cardioprotective mechanism and, therefore, a sensible therapeutic approach. Complex I reactivation is a key pathogenic characteristic of ischemia‐reperfusion injury.	Article/32

**FIGURE 7:**
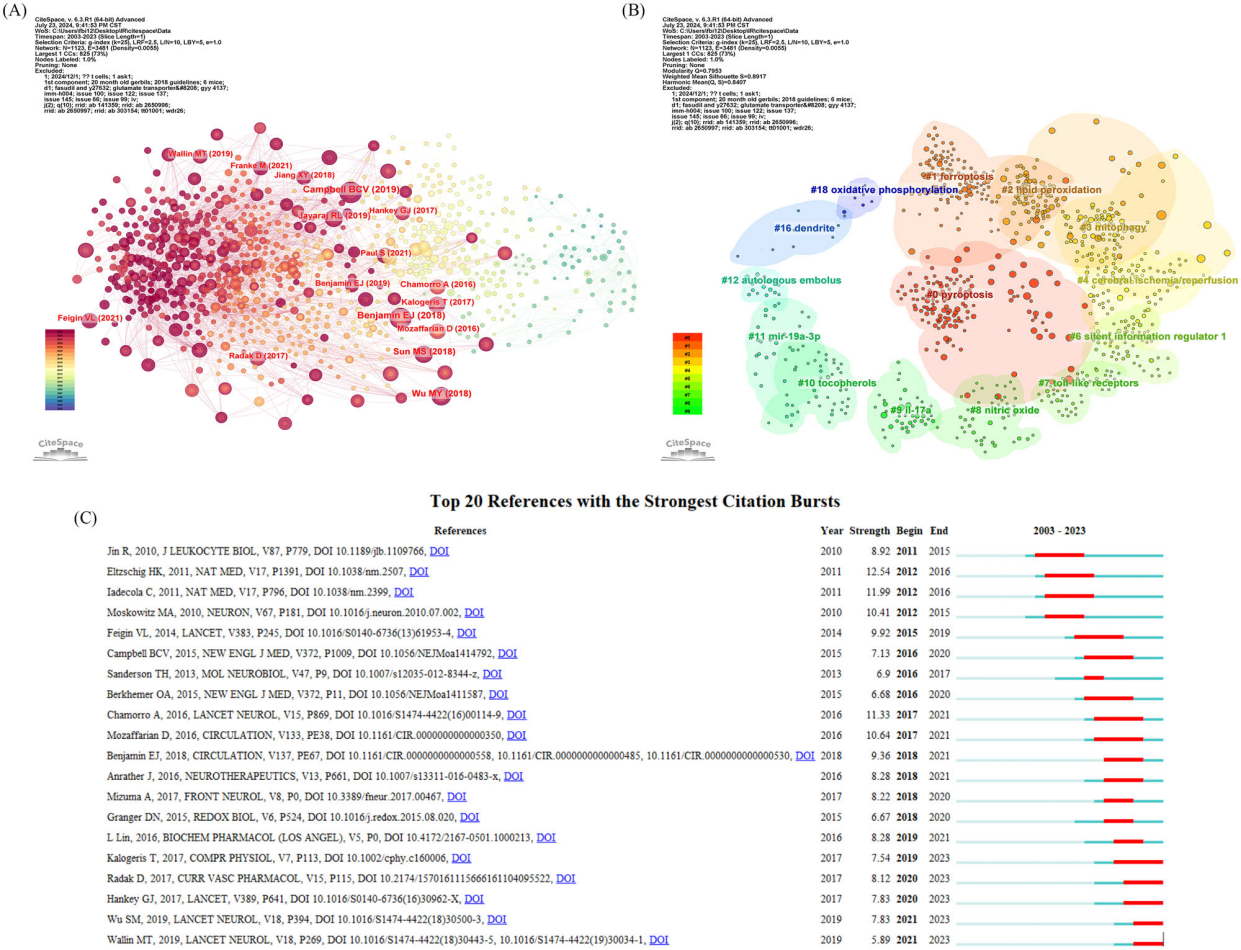
Visualization of co‐cited reference analysis. Network of co‐cited references (A); Cluster analysis of co‐cited references (B); The top 20 references with the strongest citation bursts in terms of ischemia reperfusion injury research in ischemic stroke (C).

To delve deeper into co‐cited references, CiteSpace was utilized to create a network (Figure 7A). On the basis of visualization, 19 clusters were identified, in which the modularity Q score was 0.7953 and the weighted average silhouette value S was 0.8917 (Figure 7B). Generally speaking, Q > 0.3 indicates that the keyword cluster structure is significant, and S > 0.7 is considered to be a highly efficient and convincing value (Han et al. 2023). The high modularity and silhouette values indicated the stability and validity of the clustering structure.

References that experience a surge in citations over a specific time frame are referred to as “citation burst” references. The red lines in the diagram indicate the time period during which the burst occurred. A total of 20 references are compiled in Figure 7C, which shows a significant citation burst in our study. The strength of these bursts ranged from 5.89 to 12.54, with durations spanning from 1 to 4 years. Eltzschig and Eckle (2011) conducted a study published in *Nature Medicine* titled “Ischemia and reperfusion‐from mechanism to translation” which exhibited the highest citation burst strength of 12.54 from 2012 to 2016. This was subsequently followed by Iadecola and Anrather (2011) who published “The Immunology of Stroke: From Mechanisms to Translation” in *Nature Medicine* with a citation burst strength of 11.99. The presence of recent bursts in five references indicates potential new directions in research on ischemia‐reperfusion injury studies in ischemic stroke.

### 3.7 Keywords Analysis

**FIGURE 8:**
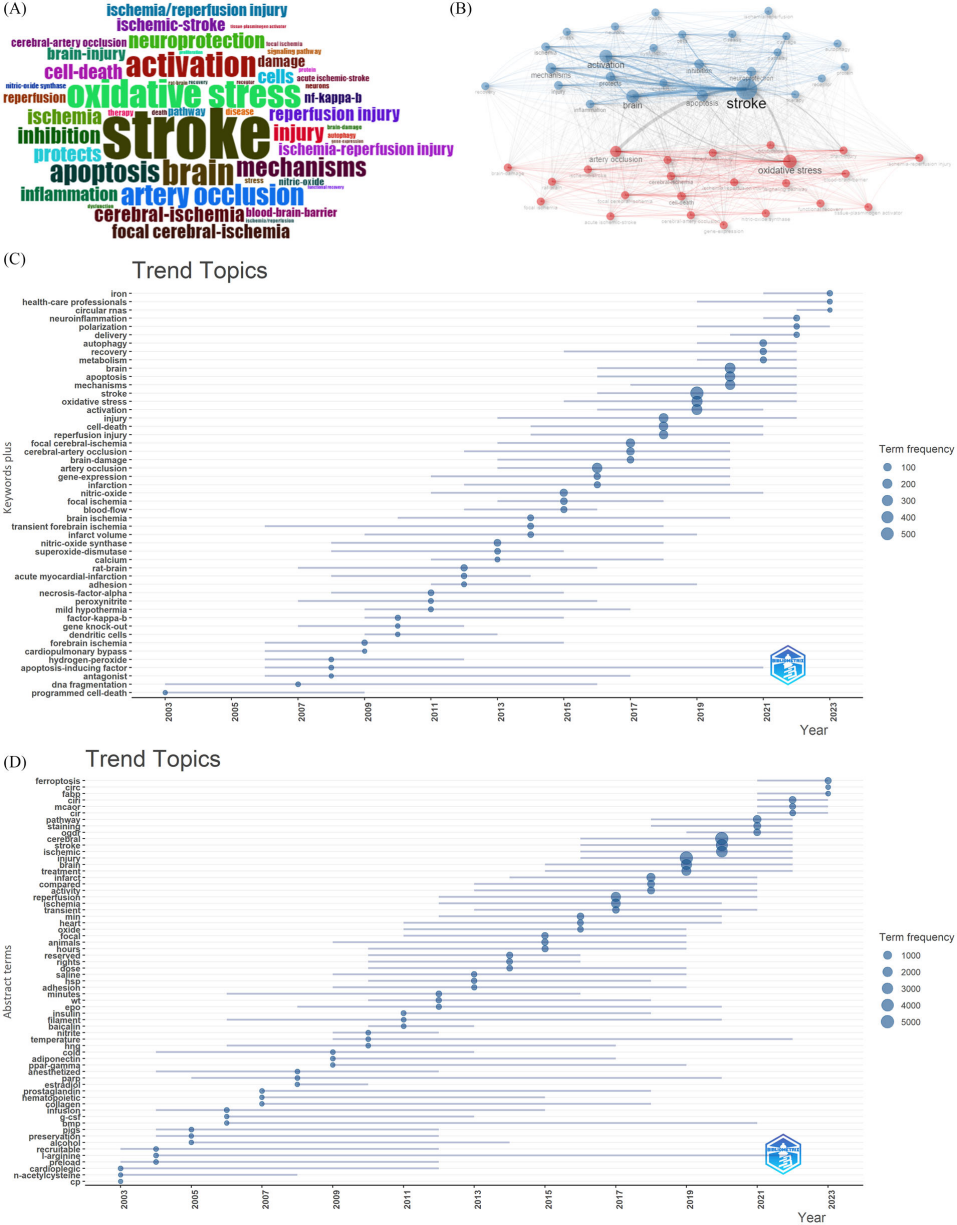
Visualization of keywords analysis. Word cloud of the most frequent keywords in ischemia reperfusion injury research in ischemic stroke (A); Keywords co‐occurrence network (B); The timeline view of research topics (C); The timeline view of abstract terms (D).

To ensure accuracy in keyword analysis, synonym merging and deletion of invalid keywords were performed. For instance, we combined the words “14‐3‐3‐proteins” and “14‐3‐3 proteins” and deleted invalid keywords such as “1 ask1” and “1st component”. Next, we highlight keywords with high frequency by visually describing them using Bibliometrix R's word cloud. The word cloud in Figure 8A displays the distribution of keywords whose font size corresponds to how frequently they occur. The 20 most frequently occurring keywords are outlined in Table 6, with the top five keywords being “stroke,” “oxidative stress,” “brain,” “activation,” and “artery occlusion.”

**TABLE 6:** The top 20 keywords in the field of ischemia reperfusion injury in ischemic stroke.

Rank	Keyword	Records	Rank	Keyword	Records
1	Stroke	560	11	Cerebral‐ischemia	171
2	Oxidative stress	311	12	Ischemia	169
3	Brain	289	13	Protects	169
4	Activation	275	14	Cell‐death	161
5	Artery occlusion	235	15	Focal cerebral‐ischemia	156
6	Apoptosis	234	16	Cells	154
7	Mechanisms	213	17	Inflammation	153
8	Injury	181	18	Reperfusion injury	152
9	Inhibition	180	19	Ischemic‐stroke	140
10	Neuroprotection	176	20	Brain‐injury	139

A keyword co‐occurrence analysis was conducted utilizing Bibliometrix R software, which identified a total of 49 keywords. As depicted in Figure 8B, the keywords were classified into two clusters. The green clusters primarily pertain to mechanisms, encompassing terms such as “apoptosis”, “neuroprotection”, “cell‐death”, and “inflammation”. The red clusters contain keywords associated with pathogenesis and etiology, such as: “oxidative stress”, “activation”, “injury”, “inhibition”, “cells”, and “protects”.

Bibliometrix R was used to examine the temporal trends of the study topics based on the frequency of all keywords in various years. It is thought to aid in the understanding of the evolution of hot spots and frontiers in this subject for researchers. The periods of keyword occurrence are illustrated by lines in Figure 8C, and the frequency of words is indicated by circles on the line. In 2023, the latest topics focused on “iron”, “health‐care professionals”, “circular RNAs”, and “polarization”. We conducted an analysis of abstract terms (Figure 8D) to uncover additional leading information. Our results indicated that “ferroptosis”, “FABP”, and “circRNA” were hot issues in 2023 and merited more investigation in subsequent research.

## 4 Discussion

Ischemia‐reperfusion injury in ischemic is a common and complex pathological process. Currently, there is no gold standard in diagnosis. The diagnosis mainly relies on clinical symptoms, signs, imaging, and laboratory indicators. Brain MRI can provide information on brain structure and function to help assess the extent of injury and locate the lesion (Pillai et al. 2009). Laboratory indicators may also aid diagnosis, for example, biochemical markers such as lactate dehydrogenase (LDH), glutamic oxalacetic transaminase (ALT), and inflammatory markers such as C‐reactive protein (CRP) may be elevated (R. Zhao et al. 2024). Studies have shown that some biomarkers may be potential biomarkers for the diagnosis of cerebral ischemia‐reperfusion injury, such as 1H NMR metabolomics (Wang et al. 2014), small molecule metabolites (Cheng et al. 2020), and a molecular probe carrying anti‐tropomyosin 4 (Yu et al. 2023).

This study utilized a thorough literature search of the Web of Science database to identify papers on ischemia‐reperfusion injury studies in ischemic stroke from 2003 to 2023. A total of 2179 eligible papers were analyzed using HistCite, VOSviewer, CiteSpace, Bibliometrix R, and Bibliometrics online analysis platform to look into global publication trends, associated institutions, authors, journals, keywords, and co‐citations in this subject. This is the first time that bibliometric metrics and visualization tools have been used to analyze the current state of ischemia‐reperfusion injury studies related to ischemic stroke, including hot spots and frontiers.

1. General information

The analysis of the results reveals a consistent upward trend in the number of publications pertaining to ischemia‐reperfusion injury studies in ischemic stroke, rising from 24 in 2003 to 258 in 2023. This trend indicates researchers are placing an increasing emphasis on this topic in recent years.

Between 2003 and 2023, China showed notable output in the field of ischemia reperfusion injury studies in ischemic stroke. However, the proportion of MCP in other countries is quite low, suggesting that international cooperation in this area still has to be strengthened. Furthermore, it is noteworthy that nine out of the ten most productive organizations are located in China, this indicates the efforts of Chinese scholars to raise the quality of scholarly work on a constant basis.

All of the top 10 authors are affiliated with institutions in China. Y. Zhang stands out as the most active (*n* = 59) and has made significant contributions to the field's advancement regarding ischemia‐reperfusion injury.

*Brain Research* emerged as the leading journal in terms of publication output among the top 10 journals, with 54 publications. Following closely behind were *Neural Regeneration Research* and *Neurochemical Research*, with 41 and 39 papers, respectively. It is remarkable that there aren't many high‐impact journals in this discipline, despite the fact that these journals publish a significant number of publications. This suggests that publishing research on ischemia‐reperfusion injury in ischemic stroke in high IF journals is still challenging. According to Braff's law, the top three core journals are *Brain Research*, *Neural Regeneration Research*, and *Neurochemical Research*.

2. Hot spots and trends

The purpose of this study is to give insight into a relevant research subject that has attracted a lot of interest from academics lately. A publication's citation count is a useful indicator of its academic impact, as highly cited publications frequently capture the main ideas in a given field of study. Through the analysis of citation counts and the identification of highly cited articles, scholars are able to discover areas of current research interest. In this study, the top 10 highly cited literature in the field were published between 2007 and 2018, the majority of which are reviews. These publications provide important insights for physicians performing research in this area and serve to summarize the developments in the field of ischemia‐reperfusion injury in ischemic stroke over different time frames.

In 2012, Kalogeris T. et al. published an article on the cell biology of ischemia‐reperfusion injury in the *International of Cell and Molecular Biology* (Kalogeris et al. 2012). The mechanism of ischemia‐reperfusion injury was described in detail. The initial ischemic insult, which is mostly influenced by the extent and length of the blood supply disruption, results in tissue damage and/or death. Subsequent damage caused by reperfusion subsequently occurs. Because of anaerobic metabolism and lactate accumulation after chronic ischemia, intracellular pH and ATP levels drop. Thus, there is an increase in intracellular and mitochondrial calcium levels (calcium overload), cell swelling and rupture, and necrotic, necroptotic, apoptotic, and autophagic mechanisms leading to cell death. ATPase‐dependent ion transport pathways also become dysfunctional as a result.

According to Eltzschig et al., severe tissue hypoxia and microvascular dysfunction are caused by an imbalance in the metabolic supply and demand in ischemic organs. The activation of innate and adaptive immune responses as well as cell death programs is further enhanced by subsequent reperfusion. Along with outlining the process of ischemia‐reperfusion harm, they additionally reviewed several approaches to treating tissue inflammation and organ dysfunction brought on by ischemia‐reperfusion. For instance, novel therapeutic modalities during ischemia and reperfusion, such as PHD inhibitors or adenosine receptor agonists, have garnered substantial support from experimental investigations of hypoxia‐elicited adaptive responses. Very powerful pharmacologic instruments to control microRNAs will soon be accessible for human medical use (Eltzschig and Eckle 2011).

In a mouse model of brain ischemia‐reperfusion injury, Gelderblom et al. (2009) described the spatiotemporal penetration of immune cell populations and their activation patterns. They discovered that the inflow of neutrophils was preceded by the increase of microglia and the infiltration of macrophages, lymphocytes, and dendritic cells (DCs) into the ischemic hemisphere. Furthermore, in the ischemic hemisphere, regulatory immune cells (natural killer T‐cells, CD_4_^−^/CD_8_^−^ T lymphocytes) accumulated. Additional understanding of poststroke immune regulation may be possible due to the unusual activation pattern and enormous expansion of antigen‐presenting cells in temporal combination with regulatory cells (Gelderblom et al. 2009).

The involvement of neuronal Toll‐like receptors in ischemic brain injury and functional impairments was investigated in a 2007 paper by Tang et al. They discovered that cerebral cortical neurons expressed TLR2 and TLR4 in response to ischemia‐reperfusion injury, and that mice lacking TLR2 or TLR4 had considerably less brain damage and neurological abnormalities following a stroke than did WT control mice. The results showed that TLR2 and TLR4 in neurons generate a proapoptotic signaling pathway that may make them susceptible to ischemia death (Tang et al. 2007).

An original article published in *Cell* by Angelina et al. examined whether P53 can activate oxidative stress‐induced necrosis. They find that p53 has a part in triggering necrosis. When exposed to oxidative stress, p53 builds up in the mitochondrial matrix and physically interacts with the PTP regulator cyclophilin D (CypD) to cause necrosis and the opening of the mitochondrial permeability transition pore (PTP). Interestingly, in the event of brain ischemia/reperfusion injury, a strong p53‐CypD complex arises. Conversely, a decrease in p53 levels or cyclosporine pretreatment of mice with this compound is linked to good protection against stroke (Vaseva et al. 2012).

One of the earliest reference citation bursts was observed in a 2010 publication by Rong Jin et al., which persisted for a duration of 4 years (2011–2015) (Jin et al. 2010), as shown in Figure 7C. The authors of this review presented an overview of the recruitment of various inflammatory cells in a time‐dependent manner after focal cerebral I/R. In addition to discussing how these cells affect ischemic brain injury, they highlighted some recent research and open‐ended concerns regarding the role of inflammatory cells in the pathophysiology of ischemic stroke. Another of the earliest reference citation bursts was reported by Michael et al. in *Neuron*, and it lasted for 3 years (2012–2015) (Moskowitz et al. 2010). This review elucidated in detail the mechanisms underlying the various treatments. The paper by Simiao Wu, identified as the most recent reference in the citation burst, provides a thorough review of advancements and hurdles made in China's stroke epidemiology, prevention, and treatment (S. Wu et al. 2019).

When it comes to swiftly capturing the distribution and evolution of hot spots in the field of ischemia‐reperfusion injury in ischemic stroke, keywords can be considered the core and soul of an article, especially when compared to references with citation outbreaks. Keywords like “stroke,” “brain,” and “artery occlusion” were excluded. The most often mentioned keywords were “oxidative stress,” “activation,” “apoptosis,” and “mechanisms,” which is in line with the investigation of the top 10 most cited papers. When considering the temporal trends of the study topics in various years, the keywords “iron”, “health‐care professionals”, “circular RNAs”, and “polarization” are the main keywords recently, indicating that clinicians are now still concerned about the mechanisms of ischemia‐reperfusion injury in ischemic stroke. Furthermore, in the abstract term analysis, the words “FABP,” “circRNA,” and “ferroptosis” were identified as hot topics in 2023 and should be further studied in future studies.

One effective therapy for ischemia‐reperfusion injury is ferroptosis (Pan et al. 2022). By effectively controlling ferroptosis in animal models of stroke, researchers have demonstrated that ferroptosis is a novel therapeutic target for stroke (Y. Xu et al. 2022). Researchers have discovered that iron death can be improved by altering the signaling route axis, which reduces cerebral ischemia‐reperfusion damage (M. Li et al. 2022). In addition, there is mounting evidence that the regulation of programmed cell death (PCDs), including iron apoptosis, is significantly influenced by mitochondrial malfunction. Neuroprotectants that target mitochondrial dysfunction have been proposed as a potentially effective treatment approach for severe secondary brain injury (She et al. 2023).

There is an increasing amount of research on circular RNA, with the main focus being on how circular RNA regulates signaling pathways to lessen brain damage following acute ischemic stroke (J. Yang et al. 2022; L. Wu et al. 2020; J. Yang et al. 2023; He et al. 2023; Z. Yang et al. 2022).

Macrophage polarization has been a hot topic in recent years. Recent studies suggest that modulation of microglial/macrophage polarization and subsequent inflammatory response may be a potential adjunct to therapeutic recirculation (X. Xu et al. 2021) (Iadecola and Anrather 2011). Therefore, many scholars have been trying to protect cerebral ischemia‐reperfusion injury by studying microglia/macrophage polarization (Luo et al. 2022; Gong et al. 2023; L. Li et al. 2023).

Fatty acid binding protein (FABP) regulates the intracellular dynamics of fatty acids, mediates lipid metabolism, and participates in signal transduction. Reperfusion therapy is the most effective treatment for acute ischemic stroke, but it often leads to secondary brain injury (Guo et al. 2021). Adipocyte fatty acid binding proteins (A‐FABP, FABP4, or aP2) have been shown to severely mediate cerebral ischemia/reperfusion (I/R) damage by exacerbating blood–brain barrier (BBB) disruption. Therefore, the study of FABP inhibitors to reduce cerebral ischemia‐reperfusion injury may be an effective therapeutic approach. Guo Q. et al. found that a novel FABP inhibitor, HY08, may be a potential neuroprotective treatment for ischemic stroke (Guo et al. 2023). In a recent study, levofloxacin was found to reduce blood–brain barrier destruction after cerebral ischemia‐reperfusion by directly inhibiting A‐FABP (S. Yang et al. 2024).

3. Limitations

Even though we used a comprehensive search method, our study has several limitations. First, the data is limited to WoSCC and may not encompass all publications within the subject. As a result, pertinent articles from other databases like PubMed, Embase, and Cochrane Library may be overlooked. In addition, this analysis ignores significant literature written in other languages and only contains English‐language texts, which could bias the selection process. Furthermore, as this study only covers literature through December 31, 2023, it's possible that recently published studies haven't received enough citations, and their usefulness is underestimated. Nonetheless, as the majority of the scientific literature on ischemia‐reperfusion injury in ischemic stroke has been incorporated into this analysis, any bias is thought to be insignificant enough to preserve the validity of the findings.

## 5 Conclusions

The objective of the current study was to investigate trends in ischemia‐reperfusion injury in ischemic stroke research by a thorough bibliometric analysis and visualization review. The consistent upward trend in the number of publications suggests researchers are placing an increasing emphasis on this topic. China has become the front‐runner in this field. The most influential institutions, journals, and authors were Capital Medical University, *Brain Research*, and Y. Zhang respectively. The predominant research focus remains on the mechanisms of ischemia‐reperfusion injury in ischemic stroke. Meanwhile, “ferroptosis”, “circular RNA”, “polarization”, and “FABP” may be the focus of future studies. This study may help novice researchers understand the advancement of ischemia‐reperfusion injury in ischemic stroke, as well as aid physicians in effectively incorporating bibliometric knowledge into their clinical practice.

## Author Contributions

**Hongyu Xu**: methodology, software, data curation, investigation, validation, formal analysis, supervision, visualization, project administration, writing – original draft. **Xinglin Lu**: investigation, writing – original draft, methodology, validation, visualization, software, formal analysis; Project administration, data curation, supervision. **Rongxing Qin**: methodology, software, data curation, investigation, validation. **Lingduo Shao**: software, investigation, validation, supervision. **Li Chen**: conceptualization, methodology, software, data curation, investigation, validation, formal analysis, supervision, funding acquisition, visualization, project administration, resources, writing – review and editing.

### Peer Review

The peer review history for this article is available at https://publons.com/publon/10.1002/brb3.70445

## Supporting information



Supporting Information

## Data Availability

The data that supports the findings of this study are available in the supplementary material of this article.
