# Supplementary material for: The Evolution of Ischemia‐Reperfusion Injury Research in Ischemic Stroke: Insights From a Two‐Decade Bibliometric Analysis
Source: Brain Behav. 2025 Apr 1;15(4):e70445. doi: 10.1002/brb3.70445 (PMC11959154; doi:10.1002/brb3.70445)
Supplement: Supplementary file 1 — Supporting Information [file BRB3-15-e70445-s001.docx]

**Supplementary Table 1**: Search strategy

|  | Search Query | Results |
| --- | --- | --- |
| #1 | ((TI=(Stroke OR Strokes OR“Cerebrovascular Accident”OR“Cerebrovascular Accidents”OR“CVA (Cerebrovascular Accident)”OR“CVAs (Cerebrovascular Accident)” OR“Cerebrovascular Apoplexy”OR “Apoplexy, Cerebrovascular”OR “Vascular Accident, Brain”OR“Brain Vascular Accident”OR“Brain Vascular Accidents”OR“Vascular Accidents, Brain”OR“Cerebrovascular Stroke”OR“Cerebrovascular Strokes”OR“Stroke, Cerebrovascular”OR“Strokes, Cerebrovascular”OR“Apoplexy”OR“Cerebral Stroke”OR“Cerebral Strokes”OR“Stroke, Cerebral”OR“Strokes, Cerebral”OR“Stroke, Acute”OR“Acute Stroke”OR“Acute Strokes”OR“Strokes, Acute”OR“Cerebrovascular Accident, Acute” OR“Acute Cerebrovascular Accident”OR“Acute Cerebrovascular Accidents” OR “Cerebrovascular Accidents, Acute”))OR AB=(Stroke OR Strokes OR“Cerebrovascular Accident”OR“Cerebrovascular Accidents”OR“CVA (Cerebrovascular Accident)”OR“CVAs (Cerebrovascular Accident)” OR“Cerebrovascular Apoplexy”OR “Apoplexy, Cerebrovascular”OR “Vascular Accident, Brain”OR“Brain Vascular Accident”OR“Brain Vascular Accidents”OR“Vascular Accidents, Brain”OR“Cerebrovascular Stroke”OR“Cerebrovascular Strokes”OR“Stroke, Cerebrovascular”OR“Strokes, Cerebrovascular”OR“Apoplexy”OR“Cerebral Stroke”OR“Cerebral Strokes”OR“Stroke, Cerebral”OR“Strokes, Cerebral”OR“Stroke, Acute”OR“Acute Stroke”OR“Acute Strokes”OR“Strokes, Acute”OR“Cerebrovascular Accident, Acute” OR“Acute Cerebrovascular Accident”OR“Acute Cerebrovascular Accidents” OR “Cerebrovascular Accidents, Acute”))OR AK=(Stroke OR Strokes OR“Cerebrovascular Accident”OR“Cerebrovascular Accidents”OR“CVA (Cerebrovascular Accident)”OR“CVAs (Cerebrovascular Accident)” OR“Cerebrovascular Apoplexy”OR “Apoplexy, Cerebrovascular”OR “Vascular Accident, Brain”OR“Brain Vascular Accident”OR“Brain Vascular Accidents”OR“Vascular Accidents, Brain”OR“Cerebrovascular Stroke”OR“Cerebrovascular Strokes”OR“Stroke, Cerebrovascular”OR“Strokes, Cerebrovascular”OR“Apoplexy”OR“Cerebral Stroke”OR“Cerebral Strokes”OR“Stroke, Cerebral”OR“Strokes, Cerebral”OR“Stroke, Acute”OR“Acute Stroke”OR“Acute Strokes”OR“Strokes, Acute”OR“Cerebrovascular Accident, Acute” OR“Acute Cerebrovascular Accident”OR“Acute Cerebrovascular Accidents” OR “Cerebrovascular Accidents, Acute”)) | 311545 |
| #2 | **((TI=(“Reperfusion Injuries”OR “Injury, Ischemia-Reperfusion”OR“Injury, Ischemia Reperfusion”OR“Ischemia-Reperfusion Injuries”OR“Injury, Reperfusion”OR“Ischemia-Reperfusion Injury”OR“Ischemia Reperfusion Injury”OR“Reperfusion Damage”OR“Damage, Reperfusion”OR“Reperfusion Damages”OR“reperfusion damage”)) OR AB=(“Reperfusion Injuries”OR “Injury, Ischemia-Reperfusion”OR“Injury, Ischemia Reperfusion”OR“Ischemia-Reperfusion Injuries”OR“Injury, Reperfusion”OR“Ischemia-Reperfusion Injury”OR“Ischemia Reperfusion Injury”OR“Reperfusion Damage”OR“Damage, Reperfusion”OR“Reperfusion Damages”OR“reperfusion damage”)) OR AK=(“Reperfusion Injuries”OR “Injury, Ischemia-Reperfusion”OR“Injury, Ischemia Reperfusion”OR“Ischemia-Reperfusion Injuries”OR“Injury, Reperfusion”OR“Ischemia-Reperfusion Injury”OR“Ischemia Reperfusion Injury”OR“Reperfusion Damage”OR“Damage, Reperfusion”OR“Reperfusion Damages”OR“reperfusion damage”))** | 30494 |
| #3 | #2 AND #1 | 2214 |
| #4 | #2 AND #1 and Article or Review Article (Document Types) | 2183 |
| #5 | #2 AND #1 and Article or Review Article (Document Types) and English (Languages) | 2179 |

**Supplementary Table 2**: Annual productions in ischemia reperfusion injury related researches in ischemic stroke.

| **Rank** | **Publication Year** | **Records** | **TLCS** | **TGCS** |
| --- | --- | --- | --- | --- |
| 1 | 2003 | 24 | 49 | 1873 |
| 2 | 2004 | 29 | 71 | 2042 |
| 3 | 2005 | 24 | 31 | 2374 |
| 4 | 2006 | 35 | 87 | 3191 |
| 5 | 2007 | 37 | 110 | 4047 |
| 6 | 2008 | 31 | 69 | 2809 |
| 7 | 2009 | 29 | 85 | 2547 |
| 8 | 2010 | 39 | 68 | 1803 |
| 9 | 2011 | 49 | 219 | 5439 |
| 10 | 2012 | 67 | 215 | 5979 |
| 11 | 2013 | 79 | 190 | 4762 |
| 12 | 2014 | 72 | 112 | 3897 |
| 13 | 2015 | 102 | 193 | 4190 |
| 14 | 2016 | 112 | 193 | 4227 |
| 15 | 2017 | 115 | 282 | 9146 |
| 16 | 2018 | 120 | 249 | 5903 |
| 17 | 2019 | 182 | 277 | 6056 |
| 18 | 2020 | 191 | 151 | 5937 |
| 19 | 2021 | 269 | 149 | 5281 |
| 20 | 2022 | 286 | 99 | 3446 |
| 21 | 2023 | 258 | 12 | 1130 |

**Supplementary Table 3**:Word collaboration ischemia reperfusion injury related researches in ischemic stroke.

| **From** | **To** | **Frequency** |
| --- | --- | --- |
| CHINA | USA | 113 |
| USA | GERMANY | 24 |
| USA | JAPAN | 18 |
| CHINA | CANADA | 12 |
| USA | ITALY | 11 |
| USA | KOREA | 11 |
| USA | UNITED KINGDOM | 11 |
| CHINA | JAPAN | 10 |
| USA | INDIA | 9 |
| CHINA | AUSTRALIA | 8 |
| CHINA | UNITED KINGDOM | 8 |
| CHINA | GERMANY | 7 |
| USA | AUSTRALIA | 7 |
| USA | SPAIN | 7 |
| GERMANY | UNITED KINGDOM | 6 |
| ITALY | UNITED KINGDOM | 6 |
| JAPAN | GERMANY | 6 |
| USA | RUSSIA | 6 |
| GERMANY | AUSTRALIA | 5 |
| UNITED KINGDOM | AUSTRALIA | 5 |
| USA | CANADA | 5 |
| USA | FRANCE | 5 |
| USA | IRAN | 5 |
| GERMANY | HUNGARY | 4 |
| ITALY | CANADA | 4 |
| ITALY | SWITZERLAND | 4 |
| USA | IRELAND | 4 |
| USA | POLAND | 4 |
| USA | SWITZERLAND | 4 |
| CHINA | IRELAND | 3 |
| CHINA | PAKISTAN | 3 |
| GERMANY | IRAN | 3 |
| GERMANY | ITALY | 3 |
| GERMANY | SWEDEN | 3 |
| INDIA | SAUDI ARABIA | 3 |
| UNITED KINGDOM | SPAIN | 3 |
| USA | FINLAND | 3 |
| USA | TURKEY | 3 |
| BRAZIL | CUBA | 2 |
| CANADA | UNITED KINGDOM | 2 |
| CHINA | ARGENTINA | 2 |
| CHINA | EGYPT | 2 |
| CHINA | KOREA | 2 |
| CHINA | SAUDI ARABIA | 2 |
| CHINA | SINGAPORE | 2 |
| GERMANY | AUSTRIA | 2 |
| GERMANY | FRANCE | 2 |
| GERMANY | ISRAEL | 2 |
| GERMANY | NETHERLANDS | 2 |
| GERMANY | SPAIN | 2 |
| GERMANY | SWITZERLAND | 2 |
| GERMANY | TURKEY | 2 |
| INDIA | UNITED KINGDOM | 2 |
| IRAN | CHILE | 2 |
| IRAN | IRAQ | 2 |
| IRAN | TURKEY | 2 |
| ITALY | ISRAEL | 2 |
| ITALY | NETHERLANDS | 2 |
| ITALY | ROMANIA | 2 |
| ITALY | TURKEY | 2 |
| JAPAN | UNITED KINGDOM | 2 |
| SPAIN | CHILE | 2 |
| SPAIN | NETHERLANDS | 2 |
| SPAIN | RUSSIA | 2 |
| SWITZERLAND | SWEDEN | 2 |
| UNITED KINGDOM | MALAYSIA | 2 |
| UNITED KINGDOM | PORTUGAL | 2 |
| UNITED KINGDOM | TURKEY | 2 |
| USA | ARGENTINA | 2 |
| USA | BELGIUM | 2 |
| USA | EGYPT | 2 |
| USA | ISRAEL | 2 |
| USA | NETHERLANDS | 2 |
| USA | PORTUGAL | 2 |
| USA | SWEDEN | 2 |
| AUSTRALIA | ARGENTINA | 1 |
| AUSTRALIA | BELGIUM | 1 |
| AUSTRALIA | BRAZIL | 1 |
| AUSTRALIA | BULGARIA | 1 |
| AUSTRALIA | IRAQ | 1 |
| AUSTRALIA | MALAYSIA | 1 |
| AUSTRALIA | THAILAND | 1 |
| BELGIUM | AUSTRIA | 1 |
| BELGIUM | HUNGARY | 1 |
| BELGIUM | NETHERLANDS | 1 |
| BELGIUM | SINGAPORE | 1 |
| BELGIUM | SWEDEN | 1 |
| BRAZIL | BULGARIA | 1 |
| BRAZIL | CHILE | 1 |
| BRAZIL | COLOMBIA | 1 |
| BRAZIL | IRAQ | 1 |
| CANADA | DENMARK | 1 |
| CANADA | EGYPT | 1 |
| CANADA | ISRAEL | 1 |
| CANADA | NETHERLANDS | 1 |
| CANADA | PAKISTAN | 1 |
| CANADA | SAUDI ARABIA | 1 |
| CANADA | SPAIN | 1 |
| CANADA | SWEDEN | 1 |
| CANADA | SWITZERLAND | 1 |
| CANADA | TURKEY | 1 |
| CHILE | COLOMBIA | 1 |
| CHINA | CYPRUS | 1 |
| CHINA | DENMARK | 1 |
| CHINA | ESTONIA | 1 |
| CHINA | FINLAND | 1 |
| CHINA | FRANCE | 1 |
| CHINA | ISRAEL | 1 |
| CHINA | MONGOLIA | 1 |
| CHINA | NETHERLANDS | 1 |
| CHINA | PORTUGAL | 1 |
| CHINA | SWEDEN | 1 |
| CHINA | THAILAND | 1 |
| CHINA | UZBEKISTAN | 1 |
| CHINA | VIETNAM | 1 |
| CUBA | COLOMBIA | 1 |
| CZECH REPUBLIC | GREECE | 1 |
| DENMARK | NORWAY | 1 |
| EGYPT | SAUDI ARABIA | 1 |
| FINLAND | ESTONIA | 1 |
| FRANCE | AUSTRIA | 1 |
| FRANCE | BELGIUM | 1 |
| FRANCE | BRAZIL | 1 |
| FRANCE | DENMARK | 1 |
| FRANCE | ISRAEL | 1 |
| FRANCE | POLAND | 1 |
| FRANCE | SPAIN | 1 |
| FRANCE | SWEDEN | 1 |
| GERMANY | ARGENTINA | 1 |
| GERMANY | CANADA | 1 |
| GERMANY | IRAQ | 1 |
| GERMANY | IRELAND | 1 |
| GERMANY | LATVIA | 1 |
| GERMANY | POLAND | 1 |
| GERMANY | ROMANIA | 1 |
| GERMANY | RUSSIA | 1 |
| GERMANY | U ARAB EMIRATES | 1 |
| INDIA | CANADA | 1 |
| INDIA | MALAYSIA | 1 |
| INDIA | NIGERIA | 1 |
| INDIA | PORTUGAL | 1 |
| INDIA | RUSSIA | 1 |
| INDIA | SWITZERLAND | 1 |
| IRAN | CANADA | 1 |
| IRAN | COLOMBIA | 1 |
| IRAN | ITALY | 1 |
| IRAN | MALAYSIA | 1 |
| IRAN | QATAR | 1 |
| IRAN | SPAIN | 1 |
| IRAN | U ARAB EMIRATES | 1 |
| IRAN | UNITED KINGDOM | 1 |
| IRAQ | BULGARIA | 1 |
| ITALY | BELGIUM | 1 |
| ITALY | CHILE | 1 |
| ITALY | EGYPT | 1 |
| ITALY | FRANCE | 1 |
| ITALY | PORTUGAL | 1 |
| ITALY | SPAIN | 1 |
| ITALY | SWEDEN | 1 |
| JAPAN | ARGENTINA | 1 |
| JAPAN | AUSTRALIA | 1 |
| JAPAN | CANADA | 1 |
| JAPAN | EGYPT | 1 |
| JAPAN | INDIA | 1 |
| JAPAN | ITALY | 1 |
| JAPAN | PORTUGAL | 1 |
| JAPAN | SPAIN | 1 |
| JAPAN | SWEDEN | 1 |
| JAPAN | SWITZERLAND | 1 |
| JAPAN | UZBEKISTAN | 1 |
| JAPAN | VIETNAM | 1 |
| KOREA | CANADA | 1 |
| KOREA | SINGAPORE | 1 |
| NETHERLANDS | IRELAND | 1 |
| NETHERLANDS | ISRAEL | 1 |
| NETHERLANDS | SWEDEN | 1 |
| POLAND | ISRAEL | 1 |
| SAUDI ARABIA | PAKISTAN | 1 |
| SAUDI ARABIA | TUNISIA | 1 |
| SINGAPORE | MEXICO | 1 |
| SLOVAKIA | CZECH REPUBLIC | 1 |
| SPAIN | BRAZIL | 1 |
| SPAIN | ISRAEL | 1 |
| SPAIN | SWEDEN | 1 |
| SPAIN | SWITZERLAND | 1 |
| SWEDEN | AUSTRIA | 1 |
| SWEDEN | ISRAEL | 1 |
| SWITZERLAND | ISRAEL | 1 |
| SWITZERLAND | NETHERLANDS | 1 |
| THAILAND | BRAZIL | 1 |
| THAILAND | BULGARIA | 1 |
| THAILAND | IRAQ | 1 |
| TURKEY | BELGIUM | 1 |
| TURKEY | CHILE | 1 |
| TURKEY | KAZAKHSTAN | 1 |
| TURKEY | PORTUGAL | 1 |
| TURKEY | QATAR | 1 |
| TURKEY | SPAIN | 1 |
| UNITED KINGDOM | BELGIUM | 1 |
| UNITED KINGDOM | BRAZIL | 1 |
| UNITED KINGDOM | CHILE | 1 |
| UNITED KINGDOM | CYPRUS | 1 |
| UNITED KINGDOM | ISRAEL | 1 |
| UNITED KINGDOM | MEXICO | 1 |
| UNITED KINGDOM | NETHERLANDS | 1 |
| UNITED KINGDOM | NEW ZEALAND | 1 |
| UNITED KINGDOM | SAUDI ARABIA | 1 |
| UNITED KINGDOM | SINGAPORE | 1 |
| UNITED KINGDOM | SWEDEN | 1 |
| UNITED KINGDOM | SWITZERLAND | 1 |
| UNITED KINGDOM | THAILAND | 1 |
| USA | AUSTRIA | 1 |
| USA | BELARUS | 1 |
| USA | BRAZIL | 1 |
| USA | CYPRUS | 1 |
| USA | DENMARK | 1 |
| USA | ESTONIA | 1 |
| USA | GREECE | 1 |
| USA | HUNGARY | 1 |
| USA | INDONESIA | 1 |
| USA | MALAYSIA | 1 |
| USA | MEXICO | 1 |
| USA | NEW ZEALAND | 1 |
| USA | NORWAY | 1 |
| USA | ROMANIA | 1 |
| USA | SAUDI ARABIA | 1 |
